# Type 2 Innate Lymphoid Cells in Allergic Disease

**DOI:** 10.2174/1573395510666140304235916

**Published:** 2013-11

**Authors:** Sean Lund, Hannah H. Walford, Taylor A. Doherty

**Affiliations:** 1 Department of Medicine, University of California, La Jolla, CA, USA; 2 Rady’s Children’s Hospital of San Diego, Division of Rheumatology, Allergy and Immunology, San Diego, CA, USA

**Keywords:** Allergy, asthma, atopic dermatitis, ILC2, nasal polyps, Type 2 innate lymphoid cells.

## Abstract

Type II innate lymphoid cells (ILC2) are a novel population of lineage-negative cells that produce high levels of Th2 cytokines IL-5 and IL-13. ILC2 are found in human respiratory and gastrointestinal tissue as well as in skin. Studies from mouse models of asthma and atopic dermatitis suggest a role for ILC2 in promoting allergic inflammation. The epithelial cytokines IL-25, IL-33, and TSLP, as well as the lipid mediator leukotriene D4, have been shown to potently activate ILC2 under specific conditions and supporting the notion that many separate pathways in allergic disease may result in stimulation of ILC2. Ongoing investigations are required to better characterize the relative contribution of ILC2 in allergic inflammation as well as mechanisms by which other cell types including conventional T cells regulate ILC2 survival, proliferation, and cytokine production. Importantly, therapeutic strategies to target ILC2 may reduce allergic inflammation in afflicted individuals. This review summarizes the development, surface marker profile, cytokine production, and upstream regulation of ILC2, and focuses on the role of ILC2 in common allergic diseases.

## INTRODUCTION

Allergic inflammation is characterized by increased Th2 cytokines including IL-4, IL-5, and IL-13 resulting in tissue eosinophilia, epithelial mucus metaplasia, and IgE production [1]. Classically, conventional CD4+ Th2 cells have been considered as the primary regulators of the allergic response through production of Th2 cytokines. However, during the past decade, a novel Th2 cytokine-producing population has been identified resulting in a paradigm shift in our understanding of allergic inflammation. Type 2 innate lymphoid cells (ILC2) were first described in 2001 by Fort *et al*. as non-B/non-T cells that produced IL-5 and IL-13 in response to IL-25 and expressed MHC class II^high^ and CD11c^dull^ [2]. Further, IL-25 administered to RAG1 knockout mice that lack B or T cells led to eosinophilia and increased tissue IL-5 and IL-13 expression suggesting that a non-B/non-T cell population was active *in vivo*. A subsequent report showed that an IL-25 responsive non-B/non-T population produced IL-5 in the lungs of mice and expressed CD45R, B220, and Thy1 (-/+), but was negative for NK1.1, Ly-6G(GR-1), CD4, CD3, and c-kit [3]. Importantly, mice deficient in NK cells, mast cells, and T/B cells mounted pulmonary eosinophilia after IL-25 administration that was not present in RAG2/γc double knockout mice, thus demonstrating the importance of γc cytokines in the development and/or maintenance of this novel population. In 2006, Fallon *et al*. described the expansion of a similar c-kit+ non-B/non-T population in the context of a helminthic infection [4]. Further, the authors demonstrated that IL-25 was required for expulsion of *N. brasiliensis* and occurred independent of B and T cells.

In 2010, three landmark independent studies further characterized the phenotype and function of Th2 cytokine producing non-B/non-T cell populations that are now termed ILC2 by consensus [5-8]. Moro *et al*. described a lineage-negative c-kit+ Sca-1+ lymphoid cell population present in the mesenteric fat of mice that produced large amounts of IL-5 and IL-13 in response to IL-33 and induced intestinal goblet cell hyperplasia after *N. brasiliensis* infection [7]. The cells were termed natural helper cells (NHC). In the same year, another report showed that a similar non-T cell population termed “nuocytes” was detected in mesenteric lymph nodes (MLN) and small intestines of IL-13 reporter mice stimulated with IL-25 and IL-33 [5]. Nuocytes expanded *in vivo* after helminth infection in an IL-25 and IL-33 dependent manner and nuocyte IL-13 was required for worm expulsion. The third report similarly described lineage-negative IL-13 producing cells termed innate helper type 2 cells (Ih2) that were increased in MLN, spleen, liver, and lung after *N. brasiliensis* infection and were sufficient to expel worms in the absence of adaptive immunity after stimulation with IL-25 [6]. Collectively, these studies demonstrated that nuocytes, natural helper cells and innate helper type 2 cells (now all termed ILC2) are novel Th2 cytokine producing cells and set the stage for a potential role for ILC2 in allergic disease. In this review, we will highlight ILC2 development, regulation, cytokine production, and potential roles in allergic diseases both in humans and in mice.

## ILC2 DEVELOPMENT

ILC2s originate from common lymphoid progenitors (CLPs) that were previously known to require inhibitor of DNA binding 2 (Id2), Notch, and the IL-7 receptor (IL-7R) that signals through the common γ chain (γc) [7, 9-11]. One report specifically showed that bone marrow natural helper cells develop from common lymphoid progenitors (CLPs) and not from either myeloid or erythroid progenitors, confirming that ILC2s are of lymphoid origin [9]. Additionally, Moro *et al*. found a near complete absence of c-kit+ Sca-1+ natural helper cells in Id2 knockout mice consistent with the requirement of Id2 for innate lymphoid cell development [7]. Several studies have since shown that mice deficient in either IL-7 or its receptor lack ILC2 [7, 12, 13].

Notch signaling was previously shown to be required for *in vitro* differentiation from CLPs into nuocytes and a recent study demonstrated that high levels of Notch signaling trigger ILC2 differentiation from human uncommitted thymic progenitor cells, and lower levels trigger a T cell program of differentiation [14, 15]. T cell factor 1 (TCF-1) is downstream of Notch signaling and was recently shown to be critical for the development of functional ILC2 [16]. In addition to Notch signaling, the transcription factor RORα was also shown to be required for nuocyte differentiation *in vitro *and for IL-25-induced nuocyte expansion and goblet cell hyperplasia *in vivo* [14]. Another report substantiated the requirement of RORα for natural helper cell development and notably showed that other ILC and Th2 cell responses were normal, suggesting more specific ILC2 regulation by RORα [17].

Recently, the master Th2 cytokine transcription factor GATA3 has been shown to play a critical role in human and mouse ILC2 development and function [18-20]. In a recent report, GATA3-deficient CLPs did not express RORα or Id2 mRNA and were not capable of producing IL-5 or IL-13 [19]. The transcription factor Gfi1 contributes to Th2 cell development by stabilizing GATA3 and a recent report evaluated the role of Gfi1 in ILC2 development and function [21, 22]. Interestingly, loss of Gfi1 led to reduced ILC2 responsiveness to IL-33 and Gfi1 knockout ILC2 expressed low levels of IL-5, but co-expressed IL-13 and IL-17, suggesting that Gfi1 contributes to the type 2 effector program of ILC2 [22]. Taken together, the development of ILC2 from CLPs depends on GATA3-induced Id2 and RORα, in addition to Notch and IL-7R signaling.

## ILC2 SURFACE MARKERS

Initial studies reported that the common surface markers expressed (though to variable levels) by Ih2, nuocytes, and NHC included CD45, Thy1.2, CD44, CD69, and c-kit (Table** 1**). Distinct phenotypic differences between these three populations exist in expression of Sca-1, T1/ST2 and IL-7Rá. These differences may reflect tissue and condition specific variations in ILC2 phenotype and/or methods of ILC2 identification. Regardless of some phenotypic distinctions, consensus has been reached to identify Ih2, nuocytes, and natural helper cells as ILC2 [8]. Human ILC2 were initially detected in the gastrointestinal tract, lung, nasal polyp tissue and peripheral blood and are defined by expression of the prostaglandin D2 (PGD2) receptor CRTH2 [23].

Notably, a separate lineage-negative population of cells termed multipotent progenitor type 2 cells (MPP2) was discovered in IL-4 reporter mice stimulated with Il-25 and was initially included along with other innate Th2 cytokine producing cells [24]. However, further work has now clearly shown that ILC2 are distinct from MPP2 that can differentiate into basophils, macrophages, and mast cells [25]. Interestingly, simultaneous activation of both ILC2 and MPP2 occurs after IL-25 exposure *in vivo* demonstrating complex parallel pathways that may contribute to type 2 inflammation.

## UPSTREAM REGULATION OF ILC2

**Fig. (1):**
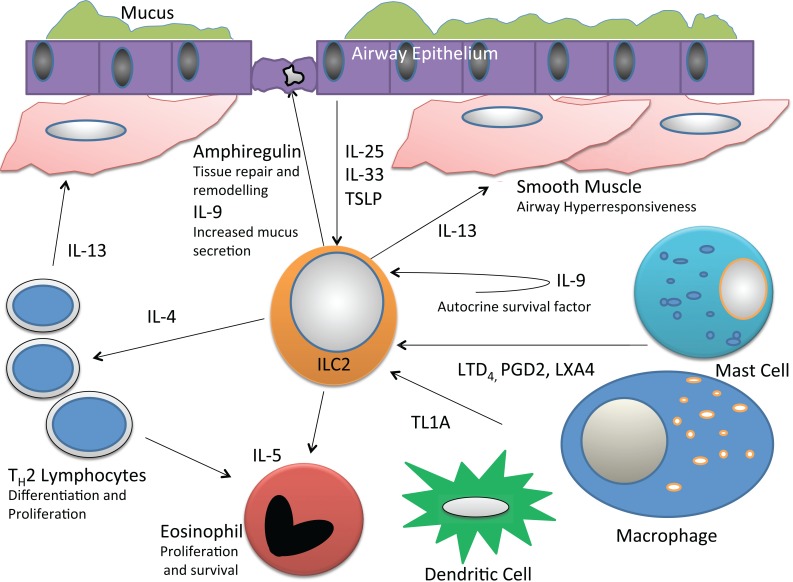
Interactions and functions of airway ILC2. ILC2 respond to epithelial cytokines TSLP, IL-33, and IL-25 and in turn produce Th2 cytokines. Additional ILC2 stimulation may occur from PGD2 and leukotriene D4 produced by mast cells and macrophages, and inhibitory signals from lipoxin A4. TL1A is also produced by dendritic cells and macrophages and can activate ILC2. ILC2 IL-5 production stimulates eosinophil activation and survival, whereas ILC2 IL-13 induces airway hyperresponsiveness, and along with IL-9, promotes mucus production. ILC2 IL-4 may contribute to Th2 cell differentiation and amphiregulin secreted by ILC2 binds to EGFR in the airway epithelium to induce repair responses.

ILC2s both respond to and are activated by a number of different molecules, including cytokines and other inflammatory mediators (Fig. **1**). Initial reports demonstrated that ILC2 were activated by IL-25 and IL-33 leading to robust Th2 cytokine production [2-7]. IL-25 (IL-17E) is a member of the IL-17 family and binds specifically to the heterodimer of IL-17RB and IL-17RA leading to activation of MAP kinase and NF-kB pathways [26]. IL-25 is secreted by many cell types including Th2 cells, eosinophils as well as epithelial cells [26]. IL-33 is released by the airway epithelium and lung macrophages, and binds to T1/ST2, a heterodimer of ST2 and the IL-1 receptor accessory protein (T1) [27-29]. IL-33 exists in a biologically active pro-form as well as a processed cleaved protein. The binding of IL-33 to T1/ST2 leads to p38 MAPK as well as NF-κB signaling [27]. Both IL-25 and IL-33 are induced in allergic lung inflammation and thus are available for potent ILC2 stimulation [13, 29-32]. One report recently demonstrated that IL-33 is more potent than IL-25 to activate IL-13 producing ILC2 and induce airway hyperresponsiveness in allergen challenged mice [33]. Consistent with this, another report showed that ST2 knockout mice, but not IL-17RB knockout mice, were impaired in allergic sensitization to house dust mite [34]. Though IL-33 appears to play a dominant role in some settings, both IL-33 and IL-25 induce different routes of activation of ILC2s that may depend on the availability of these cytokines from different cell types.

Aside from IL-25 and IL-33, more recent work has identified other mediators of ILC2 activation. TSLP is an epithelial cytokine induced in allergic inflammation and previously shown to induce conventional Th2 cell priming through the costimulatory action of OX40 ligand expressed on dendritic cells [35, 36]. Subsequently, a role for TSLP in activation of ILC2 was demonstrated as purified human peripheral blood and nasal polyp ILC2 cultured with TSLP and IL-33 displayed enhanced IL-4, IL-5, and IL-13 production above IL-33 alone [18]. TSLP has also been shown to activate mouse lung and skin ILC2 [37, 38].

Interestingly, recent reports have shown that a number of eicosanoids, arachidonic acid-derived lipid mediators, that are elevated in allergic inflammation can either directly stimulate or inhibit ILC2 [39-41]. Barnig *et al. *demonstrated that PGD2 (binds to CRTH2 expressed on human ILC2) potentiated peripheral blood ILC2 IL-13 production above IL-2, IL-25, and IL-33 *in vitro* [39]. Furthermore, this effect was lost upon the addition of lipoxin A4 (LXA4) suggesting that LXA4 has an inhibitory effect on human ILC2. In addition to PGD2 stimulation of ILC2 cytokine production, we have also detected increased human peripheral blood ILC2 chemotaxis in response to PGD2 [42]. Further, we have shown that mouse lung ILC2 express the cysteinyl leukotriene 1 receptor (CysLT1R) that is preferentially activated by leukotriene D4 [40]. Leukotriene D4 potently induced calcium influx and Th2 cytokine production from purified lung ILC2 *in vitro,* and potentiated ILC2 proliferation and lung eosinophilia in RAG2 knockout mice. The eicosanoid molecules including prostaglandins, leukotrienes and lipoxins are largely produced by macrophages, mast cells, eosinophils and dendritic cells and thus represent novel pathways through which ILC2 can be activated [41].

The TNF superfamily member TL1A binds to Death receptor 3 (DR3) and signals through TRADD and the intracytoplasmic death domain [43]. TL1A is produced by dendritic cells and macrophages in response to TLR and Fc receptor cross-linking and is also produced by T cells in response to TCR stimulation [44]. TL1A/DR3 costimulation of T cells was previously shown to be required for generation of a Th2 response and development of allergic lung inflammation in OVA models of asthma [44]. Yu *et al*. have recently reported that TL1A also directly activates ILC2 and synergizes with IL-25 *in vivo* to promote cytokine production and increase numbers of ILC2s [45]. Further, Dr3-/- mice showed an impaired ability to clear *Nippostrongylus brasiliensis *infection and had reduced ILC2 levels and lung inflammation compared with WT mice. The activation of ILC2 by TL1A provides new insight into mechanisms of ILC2 activation as TL1A is available at mucosal sites including the lung and could synergize with known ILC2 activators.

Recently, IL-9 was shown to play a critical role in ILC2 survival [46]. The authors found that IL-9 is an autocrine factor necessary for amphiregulin, IL-5, and IL-13 production, as well as eosinophil recruitment [46]. IL-9R-/- mice had reduced tissue repair responses, and this work further highlights the potential importance of ILC2 in tissue protective roles. The authors hypothesized that the antiapoptotic protein BCL3 could be conferring survival to ILC2 by inhibiting cell apoptosis associated with activation [46].

## ILC2 CYTOKINE PRODUCTION

ILC2s were initially described to produce high levels of IL-5 and IL-13 and very low levels of IL-4 in response to IL-33 *in vitro* [5, 7]. Interestingly, this cytokine pattern (high IL-5, IL-13 and low IL-4) may be more related to the effects of IL-33 as conventional T cells stimulated with IL-33 develop into IL-5 producing cells that do not produce IL-4 [47]. Subsequently, studies have shown that ILC2 stimulated with TSLP and leukotriene D4 produce IL-4 suggesting that ILC2, under certain conditions, are a source of IL-4 [18, 40]. Interestingly, lung ILC2 have recently been shown to constitutively produce IL-5 critical to eosinophil homeostasis, though IL-13 production requires IL-25 or IL-33 stimulation [48]. As stated above, ILC2 highly express the master Th2 cytokine transcription factor GATA3 that is required for ILC2 Th2 cytokine production [13, 18-20]. Though conventional Th2 cells and ILC2 share GATA3 expression, ILC2 express GATA3 in the bone marrow suggesting they are primed for Th2 cytokine production without peripheral differentiation [13, 20]. Importantly, a recent study demonstrated that TSLP further enhances GATA3 expression in human ILC2, and thus may be one mechanism of ILC2 Th2 cytokine production induced by TSLP [18].

ILC2 have also been shown to produce IL-6, IL-9, and the EGFR ligand amphiregulin [7, 32, 49, 50]. IL-9 contributes to many features of allergic asthma in animal models including airway eosinophilia and mucus production [51]. Lung ILC2 from mice challenged with the protease allergen papain were found to be a dominant source of IL-9 dependent on IL-2, and ILC2 cultured with IL-9 showed increased production of IL-5, IL-6, and IL-13 [50]. Amphiregulin is produced by ILC2 after airway administration to mice of influenza virus and the fungal allergen *Alternaria* [32, 49]. In mice receiving influenza virus, ILC2 amphiregulin promoted lung tissue repair suggesting that ILC2 may contribute to both pathogenic and healthy remodeling responses in the lung [49]. Finally, the alternatively activated macrophage enzyme Arginase I is constitutively produced by ILC2s though deletion of the Arginase I gene does not appear to inhibit cytokine production, proliferation or survival of ILC2s [52]. Overall, these reports suggest that the variable cytokine production by ILC2 during lung responses may play distinct roles depending on the inflammatory context.

## ILC2 LOCALIZATION IN TISSUES

ILC2 have been found in several different anatomical locations and have been studied in various mouse models of disease (Table **1**). Studies in mice have shown ILC2 to be present in lung, skin, mesenteric lymph nodes, bone marrow, liver, spleen, gastrointestinal tract and in mesenteric fat-associated lymphoid clusters [5-7, 9, 28, 38]. Interestingly, lung IL-5 positive ILC2 are localized to the collagen rich regions of the conducting airways, but not in alveolar spaces [48]. In humans, ILC2s have been found in nasal polyps, sinus epithelium, peripheral blood, lung, and in the gastrointestinal tract [18, 23, 49]. Thus, the extensive distribution of ILC2 in tissues suggests that ILC2 may contribute to many organ specific type 2 inflammatory responses.

## ROLES OF ILC2 IN ALLERGIC DISEASE

The roles and regulation of ILC2 in the following allergic diseases are summarized in Table **2**.

### ILC2 in Mouse Models of Asthma

Mouse models of asthma induced by allergens or viruses are characterized by features found in human asthma including airway hyperresponsivness, peribronchial inflammation, epithelial mucus production and airway remodeling. Several studies have demonstrated the importance of ILC2s in different mouse models and are also reviewed elsewhere [53, 54]. ILC2 have been shown to contribute to type 2 lung inflammatory responses and airway hyperresponsiveness (AHR) in mice infected with influenza virus and after challenge with multiple allergens including *Alternaria*, papain, house dust mite and OVA [12, 13, 28, 37, 55, 56]. The initial report by Chang *et al*. showed that ILC2 IL-13 production was required for AHR induced by influenza virus as adoptive transfer of wild type ILC2, but not IL-13 knockout ILC2, restored AHR in ILC2 depleted mice [28]. Another report demonstrated that the numbers of IL-5 and IL-13 producing lung ILC2 were shown to be equivalent to conventional cytokine producing Th2 cells in mice challenged with house dust mite [56]. Our group and others have reported that intranasal administration of the fungal allergen *Alternaria alternata *rapidly induces IL-33, activation of lung ILC2, and airway eosinophilia dependent on IL-33 signaling [12, 32]. Further, we found that ILC2 express receptors for IL-4 and IL-13 and proliferation was impaired in STAT6 deficient mice suggesting either a direct or indirect regulation of ILC2 by the IL-4/IL13/STAT6 axis. A single challenge with *Alternaria* given to mice also induces rapid cysteinyl leukotriene production dependent on STAT6 that may further regulate ILC2 activation and/or proliferation [40].

Though ILC2 appear to direct pathogenic lung responses in asthma models, there is evidence that ILC2 may promote tissue repair after viral and allergen challenges. In ILC2-depleted RAG-deficient mice infected with influenza, there was significant impairment in post infection epithelial barrier regeneration that was restored upon ILC2 transfer [49]. Importantly, the EGFR ligand amphiregulin was highly produced by ILC2 and was sufficient to induce epithelial repair after ILC2 depletion. In addition to influenza virus, *Alternaria alternata* intranasal challenges to induce ILC2 production of amphiregulin and upregulate the amphiregulin receptor EGFR supporting a role for ILC2 in tissue repair post allergen challenge as well [13]. Thus, ILC2 may have both harmful as well as beneficial effects in lung inflammation. ILC2 are activated both by epithelial-derived cytokines such as IL-25, IL-33, and TSLP as well as leukotrienes found elevated in asthma, and yet ILC2 also produce amphiregulin, an important tissue repair mediator. Airway ILC2 responses and upstream mediators are shown in Fig. (**1**). Further research is needed to elucidate the relative contribution of ILC2 during development and resolution of inflammatory responses as well as tissue repair.

### ILC2 in Human Asthma

ILC2 have not been studied extensively in human asthma, though ILC2 as well as upstream cytokines and mediators including IL-33, leukotrienes, and PGD2 have been detected in human lungs [39, 49, 57, 58]. In 2001, a report showed that human asthmatics had increased serum T1/ST2 receptor during acute exacerbation [59]. Subsequent studies have shown increased levels of IL-25, IL-33, and TSLP in patients with asthma [58, 60, 61] that all have the potential to active ILC2. In 2009, a landmark study reported the presence of a CD34+ non-B/non-T lymphocyte population found in asthmatic sputum that produced IL-5 and IL-13 in response to inhalational allergen challenge [62]. Whether or not these cells are the same as CRTH2-expressing ILC2 found later in human lungs is not clear. Taken together, these studies suggest that ILC2 may play a significant role in human asthma especially given the heterogeneity of asthma triggers apart from allergens including tobacco smoke, viruses, and ozone that could activate innate pathways.

### ILC2 in Chronic Rhinosinusitis

Chronic rhinosinusitis (CRS) is an inflammatory condition involving the mucosal lining of the nose and paranasal sinuses, estimated to affect 2% to 5% of the adult general population and is often associated with asthma, aspirin sensitivity, cystic fibrosis and allergic disease [63]. Chronic rhinosinusitis with nasal polyps (CRSwNP) represents a distinct disease entity from chronic rhinosinusitis without nasal polyps (CRSsNP). In 2011, ILC2 were first reported to be enriched in nasal polyps suggesting a potential role for these cells in CRSwNP [23]. A subsequent study identified an increased percentage of ILC2 in inflamed sinonasal mucosa (not polyp tissue) from patients with CRSwNP as compared to CRSsNP [64]. Further work has shown that ILC2 from nasal polyps produce IL-13 in a GATA3-dependent manner after culture with IL-2, IL-33 and TSLP [18]. Additionally, epithelial cells from patients with CRSwNP show high levels of mediators that regulate ILC2 including TSLP and IL-33[64, 65]. Further, TSLP enhanced the level of ILC2 GATA3 expression that may account for increased cytokine production [18]. Interestingly, ectopic expression of GATA3 by retrovirus of Lin^-^CD127^+^CD117^+^NKp44^-^CRTH2^- ^cells resulted in the induction of surface CRTH2, T1/ST2, and TSLP-R as well as IL-13 production in response to TSLP and IL-33. This work critically demonstrates the role of GATA3 in directing human ILC2 programming.

A recent consensus report suggests the importance of endotypes within CRSwNP patients defined by histopathologic features, cytokine profiles and presence of different cell types [66]. Though a large portion of nasal polyps contain high numbers of eosinophils and correlate with elevated Th2 cytokine levels, there is increasing recognition that polyps have varying cellular predominance due to distinct pathogenic mechanisms [67]. We have recently found that eosinophilic nasal polyps from allergic individuals have a higher percentage of GATA3-expressing ILC2 as compared to non-eosinophilic nasal polyps from individuals with unknown allergic status (unpublished data), thus further implicating ILC2 in the development of eosinophilic CRSwNP.

### ILC2 in Atopic Dermatitis

Atopic dermatitis is a chronic inflammatory skin condit-ion associated with skin barrier disruption, eosinophilic infiltration and high serum IgE levels. Recently, studies have reported the presence of ILC2 in healthy mouse skin [38, 68]. In one report, ILC2s have been classified as dermal ILC2 (dILC2), and differ slightly from those found in other organs as dILC2 express CD103 and lack expression of c-Kit [68]. The same study used a dsRed/IL-13 transgenic reporter and determined that the majority of cells positive for IL-13 at steady state were CD3^-^ cells, ruling out Th2 cells as primary contributors to homeostatic IL-13 production in the skin. Another group utilized a mouse model of TSLP-driven atopic dermatitis and demonstrated that ILC2 activation occurs independent of IL-33, but is dependent on TSLP receptor signaling [38]. In contrast, a separate report demonstrated that overexpression of IL-33 in the skin led to atopic dermatitis-like pathology including increased dermal eosinophils, increased Th2 cytokines and expansion of skin ILC2 [69]. Thus, the availability of specific upstream mediators of ILC2 activation likely dictates the dominant pathway resulting in ILC2 accumulation and cytokine production. Studies of ILC2 in human atopic dermatitis are limited, though similar to asthma and chronic rhinosinusitis, elevation of TSLP and IL-33 is present in human atopic dermatitis [70, 71] and one recent report demonstrated that ILC2 are increased in skin lesions of atopic dermatitis patients compared with controls [38]. Thus, ILC2 may play a role in atopic dermatitis but investigations into skin ILC2 function from diseased and non-diseased humans are needed.

## ILC2 INTERACTIONS WITH OTHER CELL TYPES

Recently, several studies have suggested that interactions between ILC2 and other cell types including CD4+ T cells, B cells, and mast cells may have meaningful effects on ILC2 levels or function. Data from the initial studies of mesenteric fat-associated ILC2 support a role for dependence on IL-2 for ILC2 cytokine production [7]. Subsequently, lung ILC2 were shown to require IL-2 for costimulation of cytokine production *in vitro* and ILC2 IL-9 production required IL-2 from adaptive immune cells *in vivo* [37, 50]. T cells are a known source of IL-2 and this suggests that adaptive immune cells are involved in the maintenance of ILC2 responses. ILC2 have also shown the capacity to produce IL-4 after stimulation with TSLP and leukotriene D4, and thus could provide an important signal leading to Th2 cell polarization [18, 40]. Interestingly, leukotriene production by dendritic cells has been shown to be required for Th2 responses to house dust mite suggesting that a leukotriene/IL-4 axis could induce Th2 cell priming and perhaps ILC2 are involved in this pathway [72]. Direct contact between ILC2 and CD4+ T cells may occur as some ILC2 populations have been shown to express MHCII [5], though the low numbers of ILC2 in tissues and lymph nodes do not support a robust pathway where ILC2 present antigen to CD4+ T cells.

ILC2 were initially described to produce IL-5 and IL-6 that induced B1 B cells to produce IgA antibody [7]. Recently, a report of a Sca-1+ Thy1+ ILC2 population in the spleens of mice that does not express IL-7R or T1/ST2, but expresses IL-18R, was shown to induce IgE production by B cells after co-culture with ILC2 and IL-18 [73]. Thus, ILC2 may amplify B cell IgE responses that are characteristic of adaptive Th2 responses. Finally, the costimulatory molecule ICOS has also been shown to be expressed by ILC2 and could lead to interactions with ICOS-ligand expressing B cells [5].

A recent report also demonstrated that ILC2 are co-localized with mast cells in the dermis [68]. The authors tracked the movements of ILC2s using intravital multiphoton microscopy and found that interactions between mast cells and ILC2 lasted as long 30 minutes. Interestingly, the authors also found that IL-9 enhanced mast cell release of IL-6 and TNFα, while IL-13 had the opposite effect and suppressed mast cell release of IL-6 and TNFα. Another report demonstrated the presence of ILC2 in close proximity to mast cells in human lung, supporting potential ILC2/mast cell interactions [39]. The interactions between ILC2 and mast cells could have important influences on shaping allergic responses as mast cell mediators including PGD2 and leukotrienes can activate ILC2 and mast cells could be reciprocally modulated by ILC2 cytokines.

## Figures and Tables

**Table 1.** Surface Marker Profiles and Tissue Distribution of Mouse and Human ILC2. Shared Surface Marker Expression As Well As Distinctions Among ILC2 Subsets are Shown

	Subtype	Tissue Localization	Surface Markers	References
Mouse	Ih2, NHC, Nuocyte shared markers		CD45, Thy1.2 (CD90.2), CD44, CD69, c-kit	[5-7, 25]
	Ih2	bone marrow, liver, lung, mesenteric lymph node, peritoneum, spleen	CD25, CD122	[6]
	NHC	fat-associated lymphoid clusters, bone marrow, lung	CD25, CD27, CD38, IL-7Rα, Sca-1, T1/ST2, GITR	[7, 9, 13, 37]
	Nuocyte	bone marrow, lung, mesenteric lymph node, spleen	CD43, CD49d, CD127, CD132, IL-7Rα, Sca-1, T1/ST2, MHC-II, Itgb7, ICOS, ICAM-1, CCR9	[5, 33, 55]
Human	ILC2	nasal polyps, sinus epithelium, peripheral blood, lung, gastrointestinal tract	CD7, CD25, CD62L, CD127, CD161, CRTH2, ST2 (IL-33R), ALX, CMKLR1, NKG2D, c-kit, DR3	[13, 18, 23, 39, 45]

**Table 2.** Regulation and Function of ILC2 in Mouse Models and Human Allergic Disease

	Allergic Disease	ILC2 Upstream Regulation	ILC2 Function	Role of ILC2 in Disease	References
Mouse	Asthma	IL-25 IL-33 LTD4 TSLP TL1A	IL-4, IL-5, IL-9, IL-13, amphiregulin	AHR, eosinophilia, mucus production, tissue repair	[12, 13, 28, 33-37, 40, 49, 55, 56]
	Atopic Dermatitis	IL-33 TSLP	IL-4, IL-5, IL-13	orthokeratosis, acanthosis, skin lesions, eosinophilia	[38, 68, 69]
Human	Asthma	IL-25 IL-33 LXA4 PGD2 TSLP	IL-13 production from peripheral blood ILC2	?	[39, 49, 57-62]
	Atopic Dermatitis	IL-33 TSLP	?	?	[38, 69, 71]
	Chronic Rhinosinusitis	IL-33 TSLP	IL-13	?	[18, 23, 64, 65]

## SUMMARY

The classic paradigm underlying allergic disease has been characterized by allergen specific Th2 cell production of IL-4, IL-5 and IL-13 leading to tissue eosinophilia, mucus production, and specific IgE production. Though the Th2 adaptive response to allergen exposure is well substantiated, recent evidence suggests that ILC2 may also contribute to pathogenic allergic inflammation. The evolutionary advantage of tissue ILC2 likely stems from an important early role in anti-parasitic immunity. In 2010, the identification of nuocytes, innate helper type 2 cells and natural helper cells as major players in type 2 immunity during helminth infections has led to investigations into ILC2 in allergic disease. ILC2, like other innate cells, do not require antigen experience and peripheral differentiation and thus could drive innate type 2 responses to viruses, tobacco smoke, and pollutants associated with asthma. Many important questions regarding the role of ILC2 in allergic diseases remain including how ILC2 contribute to ongoing allergic inflammation in the presence of intact adaptive immunity or how ILC2 can be specifically targeted therapeutically. Future studies aimed at addressing these questions will provide significant insight into mechanisms of ILC2 biology and their contributions to human disease.

## ABBREVIATIONS

AHR: = Airway hyperresponsiveness
CRTH2: = Chemoattractant receptor homologous molecule expressed on TH2 lymphocytes
DR3: = Death receptor 3
EGFR: = Epidermal growth factor receptor
GATA3: = Transacting T cell specific transcription factor 3
ICOS: = Inducible T-cell Costimulator
IgA: = Immunoglobulin A
IgE: = Immunoglobulin E
ILC2s: = Type 2 innate lymphoid cells
PGD2: = Prostaglandin D2
STAT6: = Signal transducer and activator of transcription 6
